# Interspecific Variation in Bumblebee Performance on Pollen Diet: New Insights for Mitigation Strategies

**DOI:** 10.1371/journal.pone.0168462

**Published:** 2016-12-22

**Authors:** Romain Moerman, Nathalie Roger, Roland De Jonghe, Denis Michez, Maryse Vanderplanck

**Affiliations:** 1 Evolutionary Biology and Ecology, Université Libre de Bruxelles, 50 Avenue F.D. Roosevelt, Brussels, Belgium; 2 Research Institute for Biosciences, Laboratory of Zoology, University of Mons, 20 Place du Parc, Mons, Belgium; University of California San Diego, UNITED STATES

## Abstract

Bumblebees (i.e. *Bombus* genus) are major pollinators of flowering wild plants and crops. Although many species are currently in decline, a number of them remain stable or are even expanding. One factor potentially driving changes in bumblebee distribution is the suitability of plant communities. Actually, bees probably have specific nutritional requirements that could shape their floral choices and constraint them in the current context of global change. However, most studies primarily focus on one bumblebee species at a time, making comparative studies scarce. Herein we performed comparative bioassays on three bumblebee species (i.e. *Bombus hypnorum*, *B*. *pratorum* and *B*. *terrestris*) fed on three different pollen diets with distinct nutritive content (*Cistus*, *Erica* and *Salix* pollen diets). Micro-colony performance was compared through different developmental and resource collection parameters for understanding the impact of change in pollen diet on different bumblebee species. The evidence suggests that *B*. *terrestris* is by far the most competitive species because of its performance compared to the other species, regardless of pollen diet. Our results also highlight a *Bombus* species effect as pollen diet impacts the micro-colonies in different ways according to the actual bumblebee species. Such interspecific variation in *Bombus* performance in response to a dietetic change underlines the importance of considering different bumblebee species in mitigation strategies. Such comparative studies are good advice for developing appropriate suites of plant species that can benefit threatened species while supporting stable or expanding ones.

## Introduction

Bumblebees are annual social insects mostly distributed in temperate and cold areas [1]. All 250 bumblebee species are included in the genus, *Bombus*, divided into 15 subgenera [2]. They are the dominant pollinators of many wild and crop species, providing a vital ecosystem service [3–5]. Numerous studies have documented significant bumblebee declines in both diversity and range throughout North America, Europe and Asia [6–8]. Such decreases are likely driven by multiple factors, including parasite spillover, pesticide spread, host-plant loss and global warming [7–11].

There are various bumblebee species that seem to be on the edge of extinction like *B*. *cullumanus* in Europe [12] or *B*. *franklini* in the USA [6]. However, not all bumblebee species are threatened, and many are even expanding their distribution [12]. For example, *B*. *hypnorum* was recorded new to Britain in 2001 and colonized all of England and Wales in a few years [13,14]. Another example is the buff-tailed bumblebee (*B*. *terrestris*) that was domesticated and commercialized for crop pollination [15]. This species escaped from confinement and invaded many regions in different continents (e.g. in Japan [16]; in Argentina [17]). Presently, there is little indication as to why bumblebee species differ so widely in their abundance and susceptibility to global change [18]. Several hypotheses have been put forth, like variations in climatic specialization [2,19], phenology [18], diet breadth (i.e. more or less generalist diet: e.g. [20, 21]) and particular favorite host-plants like Fabaceae [22,23]. These hypotheses are mainly based on field records and just a few experimental studies are available. For instance, the interspecific variability in nutritional performance and requirements of bumblebees remain unknown while it is definitely a key factor for understanding species conservation and potential interspecific competition [24,25].

Field studies have shown that floral resources can profoundly change in quality and quantity between close areas or over time [26]. As diet breath and favorite host plants are variable inside the genus *Bombus* (i.e. from specialists in the genus *Aconitum*, like *B*. *gerstaeckeri* to highly generalists, such as *B*. *hypnorum*; [27,28]), the interspecific competition for floral resources is therefore habitat dependent. Bumblebees with wide diet breadth, including *B*. *terrestris*, should be more competitive in a changing environment than species like *B*. *jonellus*, which forages preferentially on the Ericaceae plant family [29]. Moreover, a lack of favorite plant, without any alternative resources, induces food shortfall and leads to longer larval development [30], production of smaller or fewer individuals [31] and ejection of larvae in extreme cases [32].

Certain experimental investigations have tested the development of bumblebee colonies on different pollen diets to assess their nutritional requirements and performance (e.g. [33] on *B*. *terrestris*; [34] on *B*. *ignitus;* [30] on *B*. *terricola*). They showed that pollen source can positively or negatively impact oviposition time [33] as well as larval production [31], though they did not consider pollen traits responsible for such effects. More recent studies have associated rearing experiments with chemical analyses of the pollen diet to investigate underlying compounds. These studies, limited to a few common species, suggested that the development of colonies or the individual behavior is related to the concentration of amino acids, the presence of particular sterols or a high protein: lipid ratio [35,36, 37, 38]. As different bee species display specific nutritional requirements (e.g. sterol compounds), they could show variation that may influence their host-plant foraging patterns [25, 39, 40]. As far as we know, the study of such interspecific variability in nutritional requirements and, in turn, host-plants resource resources quality remains lacking for bumblebee species.

The aim of this work was to compare the performance of three common bumblebee species (i.e. *Bombus terrestris*, *B*. *hypnorum* and *B*. *pratorum*) on the same pollen diet under controlled conditions (i.e. bioassays). We employed an experimental setup based on micro-colonies (i.e. queenless colonies) fed on three different pollen diets to estimate brood development (e.g. brood mass) and resource collection (e.g. pollen collection). Based on the interspecific variability of floral choices observed in the field, we hypothesized that pollen diet suitability and performance was bumblebee species-dependent.

## Materials and Methods

### Bee models and pollen diets

We selected three common species of bumblebees in NW Europe: *Bombus (Pyrobombus) hypnorum*, *B*. *(Pyrobombus) pratorum* and *B*. *(Bombus) terrestris* [12]. These species display similar generalist foraging behavior but *B*. *terrestris* seems more generalist [21, 28, 41]. Moreover *B*. *hypnorum* and *B*. *terrestris* build larger colonies than *B*. *pratorum* [19]. All three species were “pollen storer” (i.e. workers store pollen in cells before feeding the larvae), making their breeding under the same experimental conditions possible [18].

We considered three pollen diets for the bioassays, namely *Cistus*, *Salix* and *Erica*. We used honeybee pollen loads purchased from the company “Pollen Energie” (St Hilaire de Lusignan, France). Worker honeybees forage on pollen from various resources but each worker individually specializes in one pollen resource, making a monofloral pollen load. The target pollen species may be differentiated based on their color. We double-checked the uniqueness of the plant species composition of the blends by analyzing the pollen grain morphology under a light microscope (Leitz at ×400 magnification). Pollen of *Sali*x has been previously described as an excellent resource for *B*. *terrestris* colony development (18.6% of total amino acid content) while *Cistus* pollen had a rather negative impact on colony development (13.5% of total AA content), the pollen of *Erica* showing intermediate results (13.5% of total AA content) [see 35 for description of chemical characteristics of these blends].

### Rearing setup

We collected newly-emerged wild queens of *B*. *hypnorum* and *B*. *pratorum* in the Spring of 2014 and 2015 in the areas of Brussels and Mons (Belgium). *B*. *hypnorum* and *B*. *pratorum* queens were abundant on *Salix caprea* and *Ribes sanguineum*. Sampling of *B*. *hypnorum* was completed with full nests found in the wild in the areas of Westerlo (Belgium) and Arlon (Luxembourg) (e.g. in old bird boxes). *B*. *terrestris* colonies were provided by Biobest bvba (Westerlo, Belgium). All colonies of the three species were reared with *Salix* pollen in a dark room at 26°C and 65% relative humidity until worker emergence. The samples did not involve endangered or protected species. No specific permits were required for the described field studies as insect collection did not occur in privately owner or protected locations.

We randomly collected four two-day-old workers from colonies to constitute micro-colonies (i.e. queenless colonies) following the method developed by Regali and Rasmont [42]. Each micro-colony was reared in a plastic box (8 x 16 x 16 cm). After a few days, one worker became dominant and began to lay male eggs [33]. We removed from the analyses micro-colonies without brood development. Such a method using queenless *B*. *terrestris* micro-colonies for testing the nutritive value of pollen diets was previously shown to be an appropriate estimate of queenright colony development at least under laboratory conditions [43]. We were able to produce 30 micro-colonies of *B*. *terrestris*, 25 of *B*. *pratorum* and 19 of *B*. *hypnorum*.

While the usual rearing temperature was 28°C or 30°C for bumblebees [35], the 74 micro-colonies were reared in the same dark room at 26°C and 65% relative humidity. This lower temperature was selected after primary testing demonstrated a better development of colonies and micro-colonies of *B*. *pratorum* and *B*. *hypnorum* without significant influence on *B*. *terrestris* colony development. All micro-colonies were fed *ad libitum* during a 21-days period with pollen provided as candy (see below) and inverted sugar syrup (BIOGLUC, Biobest, Westerlo, Belgium) provided by capillary tube placed under the micro-colony and in contact with a stock of syrup. Preliminary test did not reveal any significant evaporation or condensation on the stock of syrup in the experimental conditions.

To facilitate diet manipulation, the same ratio of mass pollen was mixed with inverted sugar syrup (90% and 10% w/w, respectively) to form candies. New pollen candy was provided every two days to avoid alteration of pollen content.

### Bumblebee performance

Several parameters were considered to evaluate micro-colony development (adapted from [44]): (i) total pollen collection (i.e. fresh mass of pollen consumed and stored); (ii) total syrup collection (i.e., mass of syrup consumed and stored); (iii) mass of offspring (total larvae and pupae); (iv) mean pupal mass; (v) number of eggs; (vi) number of larvae; and (vii) number of pupae. All weighed parameters (i.e., brood, pollen or syrup) were standardized by the mass of the four workers’ abdomens (i.e. estimator of total body mass) from each micro-colony to cancel the potential effect of worker activities linked to their size (i.e. consumption and brood care) [45]. The pollen efficacy parameter was estimated as the total mass of offspring divided by total pollen collection [44]. Additionally, pollen and syrup collection per gram of offspring along with pollen dilution (i.e. pollen collection/syrup collection) were calculated as indicators of performance.

Furthermore, we considered the fat body content of workers as an indicator of individual condition because fat body is involved with the immune system (i.e. synthesis of proteins with antimicrobial activities) and in nutrient storage (i.e. proteins, lipids and carbohydrates) [46]. The abdomens of workers were dried at 70°C over three days and then weighed. These dried abdomens were then placed in 2 mL of diethyl ether for 24 hours. After rinsing twice with diethyl ether, these abdomens were placed seven days at 70°C and weighed [47]. Mass difference between the two weights was used as the parameter after standardization by the initial weight to avoid biases linked to worker size.

### Statistical analyses

Generalized linear mixed models (GLMMs) were utilized to test the influence on the different parameters (i.e. resource collection, developmental parameters, fat body) of two fixed categorical variables related to the *Bombus* species (three levels: *B*. *hypnorum*, *B*. *pratorum* and *B*. *terrestris*) and diet (three levels: *Cistus*, *Erica* and *Salix*), including the year (two levels: 2014 and 2015) as a random factor (“lmer” function, R-package lmerTest). Prior to these analyses, percentage data (i.e. fat body content) were arcsine-transformed to achieve variance stabilization. Normality of the residuals and overdispersion of the data were verified (p > 0.05). Data were transformed when assumption violation occurred (i.e. log- or rank-transformed). The effects of fixed and random factors (i.e. analysis of variance (ANOVA) and difference of least-squares means) were assessed using the step function implemented in the package stats [48]. Data were visualized on plots of means and boxplots for parametrical and non-parametrical data, respectively. All analyses were performed in R version 3.0.2 [48].

Brood compositions (i.e. proportion of eggs, larvae and pupae) were compared using permutational multivariate analysis of variance (perMANOVA) based on the Bray-Curtis dissimilarity index and 999 permutations (“adonis” command, R-package vegan [49]). Prior to the perMANOVA, the multivariate homogeneity of the within-group covariance matrices was inspected using the “betadisper” function implementing Marti Anderson’s testing method. Distinct perMANOVAs were performed using the *Bombus* species, diet or their interaction as factor variables to detect significant differences in the dynamics of micro-colony development. Data were visually assessed on a histogram. All analyses were performed in R version 3.0.2 [48]. All results are presented as mean ± SD.

## Results

**Fig 1:**
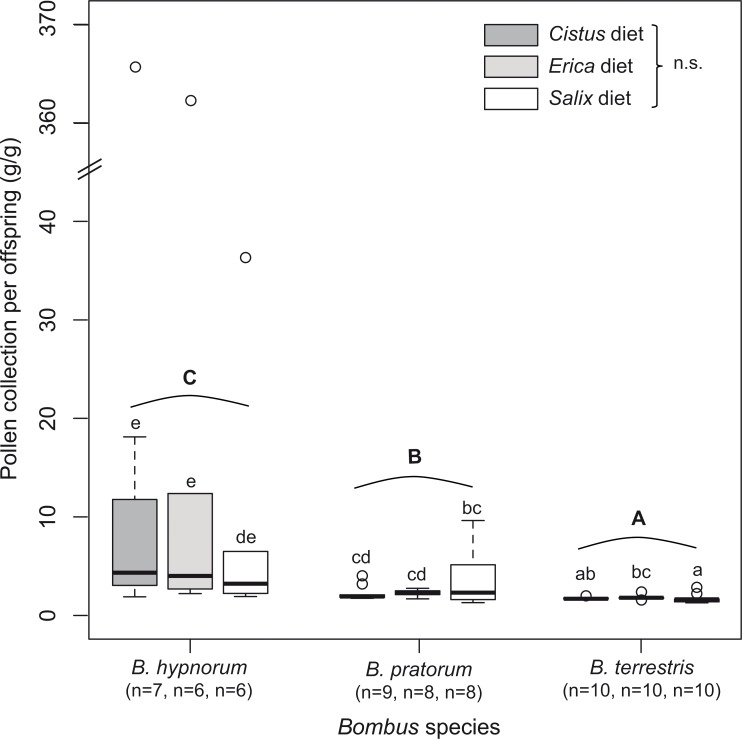
Pollen collection per offspring for micro-colonies of the three bumblebee species (*B*. *hypnorum*, *B*. *pratorum* and *B*. *terrestris*) reared on the three pollen diets (*Salix*, *Cistus* and *Erica*). Differences across species were significant (GLMMs: F_2,64.09_ = 31.39, p < 0.001; see Results). Majuscule letters indicate interspecific significant differences and minuscule letters indicate intraspecific significant differences.

Pollen collection was species dependent (F_2,65_ = 9.94, *p* < 0.001) with a lower collection for *B*. *hypnorum* (37.11 ± 14.14 g) compared to *B*. *pratorum* (51.81 ± 13.61 g) and *B*. *terrestris* (53.41 ± 11.60 g) (S1 Table). Regardless of the *Bombus* species, pollen collection did not depend on pollen diet (F_2,65_ = 0.23, *p* = 0.796) and no effect of factor interactions (i.e. diet: *Bombus* species) was detected. Considering pollen collection per gram of offspring (i.e. reciprocal of pollen efficacy), statistical analyses determined significant differences between all species with *B*. *terrestris* displaying the lowest median pollen collection per gram of offspring (1.71 g/g offspring), *B*. *hypnorum* the largest (3.93 g/g offspring) and *B*. *pratorum was* intermediate (2.10 g/g offspring) (F_2,64.09_ = 31.39, *p* < 0.001) (Fig 1). Although no main diet effect was detected, micro-colonies of *B*. *terrestris* fed on the *Erica* diet displayed a larger pollen collection per gram of offspring, significantly different from the *Salix* diet (t = 2.18, df = 64, *p* = 0.033) (Fig 1).

Syrup collection was not impacted by diet (F_2,62.99_ = 2.49, *p* = 0.091) but depended on the *Bombus* species (F_2,63.01_ = 54.14, *p* < 0.001), with a lower syrup collection for *Bombus terrestris* (433.23 ± 125.16 g) compared to *Bombus hypnorum* (692.38 ± 141.27 g) and *Bombus pratorum* (819.73 ± 237.46 g) (S1 Table) seen. Syrup collections per gram of offspring were significantly varied between species. *B*. *terrestris* displayed the lowest median syrup collection per gram of offspring (13.22 g/g offspring), *B*. *hypnorum* the largest (68.34 g/g offspring) and *B*. *pratorum* was intermediate (32.94 g/g offspring) (F_2,64_ = 70.91, *p* < 0.001) (S1 Table). No diet effect or factor interactions (i.e. diet: *Bombus* species) was detected. Syrup collection weighted by pollen collection (i.e. pollen dilution) highlighted that workers of *B*. *terrestris* collected a significantly lower amount of syrup per gram of pollen versus the other *Bombus* species, regardless of pollen diet (F_2,62.72_ = 111.01, *p* < 0.001).

**Fig 2:**
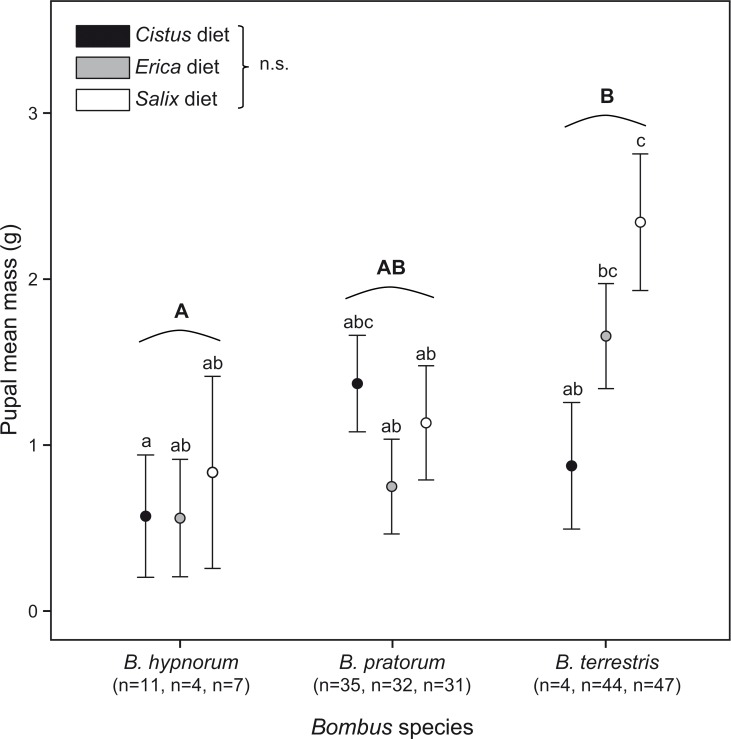
Pupal mean mass for micro-colonies of the three bumblebee species (*B*. *hypnorum*, *B*. *pratorum* and *B*. *terrestris*) reared on the three pollen diets. Differences across diets were significant for *B*. *hypnorum* as it produced smaller pupae than *B*. *terrestris* (GLMMs: F_2,64.32_ = 4.78, *p* = 0.012; see Results). Micro-colonies of *B*. *terrestris* produced higher mass pupae on *Salix* diet versus the *Cistus* diet (t = -3.10, df = 64, *p* = 0.003). Majuscule letters indicate interspecific significant differences and minuscule letters indicate intraspecific significant differences.

Brood masses (i.e. larval and pupal masses) were significantly different between the *Bombus* species (F_2,64.07_ = 26.47, *p* < 0.001), though no diet effect was observed (F_2,64.01_ = 0.29, *p* = 0.753). Multiple pair-wise comparisons revealed that *B*. *terrestris* produced the heaviest broods (31.54 ± 9.23 g), *B*. *hypnorum* the lightest (10.88 ± 9.24 g) and *B*. *pratorum* broods were intermediate (23.66 ± 10.41 g) (S1 Table). Certain differences were noted for the mean pupal mass between the different species as *B*. *hypnorum* produced smaller pupae than *B*. *terrestris* (F_2,64.32_ = 4.78, *p* = 0.012) (Fig 2). As pupal masses were weighted by worker masses prior to statistical analyses, such differences were associated with bumblebee performance and not to species-dependent traits. Despite no significant diet impact on mean pupal mass, statistical analyses revealed that, on average, pupae of *B*. *terrestris* fed on the *Salix* diet exhibited a higher mass than those fed on the *Cistus* diet (t = -3.10, df = 64, *p* = 0.003) (Fig 2). While the same trends were observed for *B*. *hypnorum*, the opposite seemed to take place with *B*. *pratorum* with larger pupae produced in micro-colonies fed on the *Cistus* diet (Fig 2).

**Fig 3:**
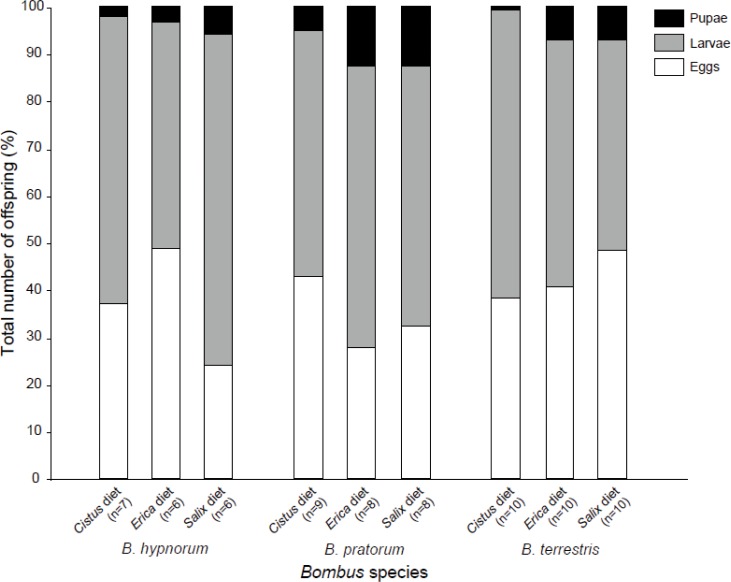
Percentage of pupae, larvae and eggs in the total number of offspring for the three species (*B*. *hypnorum*, *B*. *pratorum* and *B*. *terrestris*) reared on the three pollen diets (*Salix*, *Cistus* and *Erica*). No differences were observed in the dynamics of growth (perMANOVA: F_2,71_ = 1.00, *p* = 0.38).

A detailed study of the number of specimens uncovered that broods of *B*. *hypnorum* had less larvae (F_2,64.29_ = 19.18, *p* < 0.001) and pupae (F_2,65_ = 4.59, *p* = 0.014) compared to other *Bombus* species as well as fewer eggs versus *B*. *terrestris* (F_2,65_ = 5.72, *p* = 0.005) (S1 Table). However, micro-colonies of *B*. *hypnorum* did not demonstrate significantly slower dynamics in comparison to the other species (perMANOVA, F_2,71_ = 1.00, *p* = 0.38) as their broods possessed similar proportions of eggs, larvae and pupae to the other *Bombus* species (Fig 3). Although no significant main diet effect was found, micro-colonies of *B*. *pratorum* fed on the *Cistus* diet had a greater offspring production (t = 2.26, df = 64.1, p = 0.027), especially larval production (t = 2.49, df = 64.1, p = 0.015), than those fed on the *Salix* diet (S1 Table).

**Fig 4:**
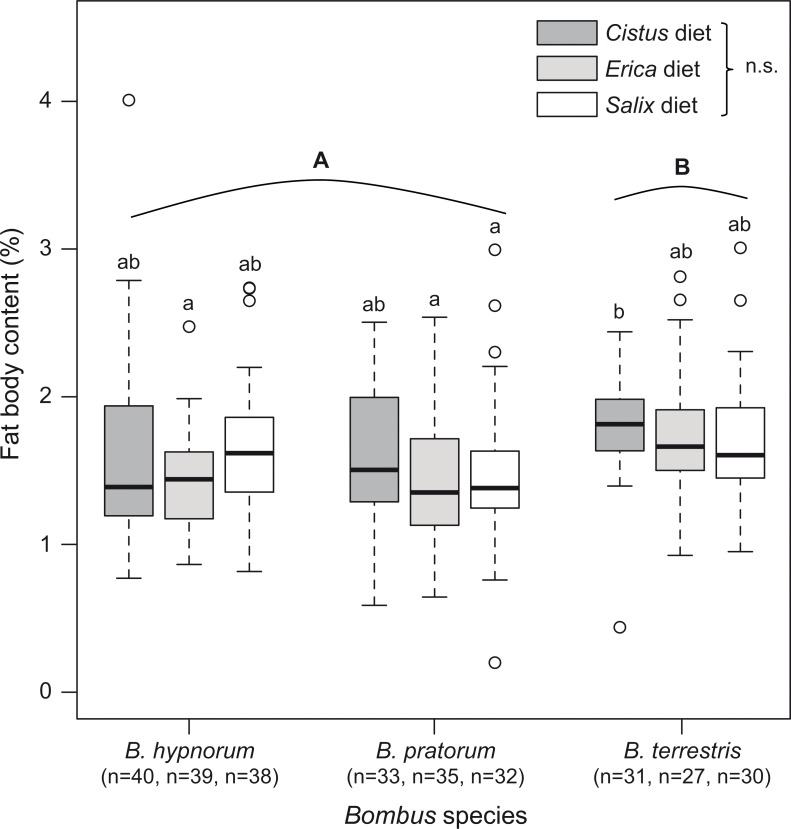
Fat body content of the three bumblebee species (*B*. *hypnorum*, *B*. *pratorum* and *B*. *terrestris*) reared on the three pollen diets. Differences across diets species were significant for *B*. *terrestris* (GLMMs: F_2,296_ = 10.11, *p* < 0.001; see results). No impact from diet on the fat body content was detected (F_2,296_ = 2.29, *p* = 0.103). Majuscule letters indicate interspecific significant differences and minuscule letters indicate intraspecific significant differences.

Workers of *B*. *terrestris* displayed a significantly larger median fat body content (1.68%) compared to *B*. *hypnorum* (1.48%) and *B*. *pratorum* (1.42%) (F_2,296_ = 10.11, *p* < 0.001) (S1 Table and Fig 4). However, the fat body content of the three species was not impacted by the diet (F_2,296_ = 2.29, *p* = 0.103) (S1 Table and Fig 4).

## Discussion

### Bombus species-dependent colony performance

We found a significant difference in the colony development of the three studied species (i.e. *B*. *hypnorum*, *B*. *pratorum* and *B*. *terrestris*) while fed on the same pollen diets under the same conditions. By far, *B*. *terrestris* had the highest performance (i.e. pollen collection per gram of offspring) needing two-fold less pollen and six-fold less syrup to produce the same brood mass as *B*. *hypnorum*. This species also showed the highest mean pupal mass as well as the greatest percentage of fat body. These traits are obviously positively correlated with colony fitness as: (i) higher performance means that workers need to collect less resources (i.e. spend less energy in foraging) to produce an equivalent mass of offspring; (ii) larger workers can collect more pollen and nectar resources than smaller ones [50]; and (iii) well-developed fat body helps to better resist parasites and diseases [51, 52]. Actually, its foraging behavior (i.e. highly polylectic with more than 20 host plants foraged) coupled with its high performance (i.e. high colony development regardless of pollen diet) allows *B*. *terrestris* to quite easily incorporate novel hosts (i.e. being ecological opportunists with large-scale plasticity with regards plant use) when compared to other bumblebee species [21, 23, 28]. Overall, these species traits may partly explain why *B*. *terrestris* is so competitive in its native range and exhibits a high capacity for invading new regions. Our results reinforce the argument of controlling the international market of this species in non-native areas [53].

Relative performance of experimental queenless micro-colonies could be different from natural queenright colonies; and the observed interspecific variations could be associated to a greater plasticity of *Bombus terrestris* worker to micro-colony organization and experimental conditions. However, Tasei and Aupinel [43] showed that micro-colonies of *Bombus terrestris* displayed similar performance than colonies of *Bombus terrestris* on the same diet. Moreover, the organization of micro-colony (e.g. one dominant laying worker with four non-laying workers) is quite similar to the organization of any young bumblebee colony (e.g. one laying queen with a first reduced batch of non-laying workers). Actually the artificiality of bumblebee queenless micro-colony is quite low compared to the artificiality of a group of non-laying honey bee workers [42]. Overall, these arguments support using micro-colony in experimental design to compare the performance of different bumblebee species although such experimental design remains a simplification of a mature bumblebee colony.

### Pollen diet-dependent colony performance

As previously underscored [33, 44, 54], bumblebee colony growth was significantly affected by pollen sources. As a matter of fact, the development of *B*. *terrestris* was impeded (e.g. lower mean pupal mass) on *Cistus* pollen compared to *Salix* pollen. Interestingly, the performance of *B*. *hypnorum* micro-colonies were similar on the three pollen diets, the number of larvae of *B*. *pratorum* being even higher with *Cistus* pollen than *Salix* pollen. Such interspecific variation in micro-colony performance from varied pollen diets could be explained by different species-dependent abilities to physiologically cope with pollen traits (i.e. structure and chemical composition). Pollen nutritional quality varies widely among plant species both quantitatively (i.e., ranging from 2–60% protein and 1–20% lipids by weight) [55, 56] and qualitatively (i.e. difference in sterol and amino acid profiles) [23, 52]. In this way, *Cistus* pollen has been described with a higher relative concentration of 24-methylenecholesterol than that of *Salix* [35], which could account for the higher performance of *B*. *pratorum* on this diet. Currently, experimental studies are still necessary to better understand the various nutritional requirements of wild bumblebee species and then establish their optimal diets. However, only three species have been herein considered, and our results already emphasize the interspecific variability of bumblebee performance on different pollen diets. Evidence from the experimental bioassays conducted on *B*. *terrestris* may not be extrapolated to other species, and this needs to be considered for mitigation strategies, particularly in terms of developing nutritionally-balanced plant communities.

### Bumblebee conservation

Attention has been paid worldwide to bumblebee conservation because they are the dominant pollinators of many wild and crop plant species [57]. An important component of mitigation strategies is increasing the availability of floral resources, though resource quality has been poorly taken into account until now (see review in [25]). Our results indicate that a generalist and dominant species like *B*. *terrestris* seems able to develop well on a wide diversity of pollen resources, producing an abundant offspring. Consequently, a shift in host-plant resources probably does not impact its conservation significantly [23]. Yet, more selective bumblebee species with lower plasticity and performance could be impacted by such a resource shift [24]. Therefore, mitigation strategies should consider this interspecific variability to optimize the selection of host-plant resources. Special attention has to be paid to what constitutes appropriate suites of plant species that benefit threatened species (i.e. those in decline) while supporting generalist species with higher performance (i.e. stable or in expansion) [25].

## Supporting Information

S1 Table Micro-colonies development. Parameters of bumblebee micro-colonies measured for three species (*B*. *hypnorum*, *B*. *pratorum* and *B*. *terrestris*) reared on the three pollen diets (*Cistus*, *Salix* and *Erica*).
(DOCX)
