# Supplementary material for: Interspecific Variation in Bumblebee Performance on Pollen Diet: New Insights for Mitigation Strategies
Source: PLoS One. 2016 Dec 22;11(12):e0168462. doi: 10.1371/journal.pone.0168462 (PMC5179047; doi:10.1371/journal.pone.0168462)
Supplement: S1 Table — Parameters of bumblebee micro-colonies measured for three species (B. hypnorum, B. pratorum and B. terrestris) reared on the three pollen diets (Cistus, Salix and Erica). (DOCX) [file pone.0168462.s001.docx]

**S1 Table. Micro-colonies development**. Parameters of bumblebee micro-colonies measured for three species (*B. hypnorum*, *B. pratorum* and *B. terrestris*) reared on the three pollen diets (*Cistus*, *Salix* and *Erica*). Results are presented as minimum – maximum (mean).

| Parameters |  | *Bombus hypnorum* | | | | |  | *Bombus pratorum* | | | | |  | *Bombus terrestris* | | | | |
| --- | --- | --- | --- | --- | --- | --- | --- | --- | --- | --- | --- | --- | --- | --- | --- | --- | --- | --- |
|  |  | *Cistus* diet  (n =7) |  | *Erica* diet  (n =6) |  | *Salix* diet  (n =6) |  | *Cistus* diet  (n =9) |  | *Erica* diet  (n =8) |  | *Salix* diet  (n =8) |  | *Cistus* diet  (n =10) |  | *Erica* diet  (n =10) |  | *Salix* diet  (n =10) |
| Syrup collection (g) |  | 545-989  (659) |  | 565-907  (739) |  | 509-936  (684) |  | 437-992  (721) |  | 622-1223 (851) |  | 552-1347 (900) |  | 287-543 (404) |  | 324-763 (478) |  | 314-561  (416) |
| Syrup collection (g/g offspring) |  | 24.4-7619 (1253) |  | 40-10153.8 (1783.2) |  | 40.2-561.4 (144.7) |  | 20.7-98  (36.1) |  | 28.7-59.5 (41.5) |  | 20.9-138.7 (55.6) |  | 10.5-20.3 (14) |  | 10.5-27.9 (14.5) |  | 8.8-18.5 (14.4) |
| Pollen collection (g) |  | 12.1-64.3 (38.7) |  | 24.9 46.5 (35.3) |  | 26.8-48.0 (37) |  | 18-78.6 (51.1) |  | 36.4-60.3 (47.5) |  | 34.6-79.4 (56.9) |  | 40.1-64.3 (49.6) |  | 50.1-77.9 (60.9) |  | 29.7-72.3 (49.7) |
| Pollen collection (g/g offspring) |  | 1.9-365.7 (57.4) |  | 2.2-362.3 (64.6) |  | 1.9-36.3 (8.9) |  | 1.7-4 (2.3) |  | 1.7-2.7 (2.3) |  | 1.3-9.6 (3.6) |  | 1.5-2 (1.7) |  | 1.5-2.4 (1.8) |  | 1.3-2.8 (1.7) |
| Pollen dilution (g/g) |  | 10.1-45 (21.9) |  | 18-28 (21.8) |  | 13.8-25.5 (19.1) |  | 11-19.7 (15) |  | 13.6-24.3 (17.9) |  | 13.6-21.1 (15.7) |  | 6.1-11.5 (8.2) |  | 5.9-11.7 (7.8) |  | 6.4-11.2 (8.5) |
| Number of eggs |  | 0-41 (16) |  | 2-24 (11) |  | 0-13 (5) |  | 7-61 (30) |  | 0-46 (16) |  | 0-53 (17) |  | 6-42 (21) |  | 0-40 (26) |  | 0-60 (33) |
| Number of larvae |  | 1-44 (14) |  | 1-29 (13) |  | 2-22 (7) |  | 16-41 (32) |  | 6-43 (21) |  | 3-37 (18) |  | 23-40 (31) |  | 22-35 (29) |  | 4-45 (22) |
| Number of pupae |  | 0-7 (2) |  | 0-3 (1) |  | 0-4 (1) |  | 0-8 (4) |  | 0-11 (4) |  | 0-10 (4) |  | 0-1 (1) |  | 0-9 (0) |  | 0-10 (5) |
| Number of offspring |  | 1-48 (15) |  | 1-32 (13) |  | 2-22 (8) |  | 16-49 (36) |  | 12-43 (25) |  | 3-47 (22) |  | 24-40 (31) |  | 22-42 (33) |  | 6-41 (27) |
| Mass of larvae (g) |  | 0.1-21.3 (9) |  | 0.1-15.7 (8.2) |  | 0.9-13.7 (7.9) |  | 13.9-29 (18.9) |  | 6.5-23.5 (14.8) |  | 8.2-25.1 (17.4) |  | 18.9-38.1 (28.4) |  | 10.1-49.9 (25) |  | 6.8-48.1 (21.1) |
| Mass of pupae (g) |  | 0-13.9 (3.1) |  | 0-5 (1.1) |  | 0-13.7 (3.1) |  | 0-16.2 (6.8) |  | 0-15.2 (6) |  | 0-15.6 (6.9) |  | 0-3.2 (0.9) |  | 0-20.1 (8.9) |  | 0-17.8 (10.4) |
| Mass of offspring (g) |  | 0.1-29.4 (12.1) |  | 0.1-20.7 (9.3) |  | 0.9-23.3 (10.9) |  | 4.5-45.2 (25.6) |  | 16-26.9 (20.8) |  | 8.2-49 (24.3) |  | 21-40.2 (29.3) |  | 22.5-42.7 (33.9) |  | 10.4-54.4 (31.5) |
| Diet efficacy (g/g) |  | 0-0.5 (0.2) |  | 0-0.4 (0.2) |  | 0-0.5 (0.3) |  | 0.2-0.6 (0.5) |  | 0.4-0.6 (0.4) |  | 0.1-0.8 (0.4) |  | 0.5-0.6 (0.6) |  | 0.4-0.6 (0.5) |  | 0.3-0.8 (0.6) |
| Fat body content (%) |  | 0.8-2.6 (1.6) |  | 0.9-2.5 (1.4) |  | 0.8-2.7 (1.7) |  | 0.6-2.3 (1.6) |  | 0.6-2.5 (1.4) |  | 0.2-3 (1.5) |  | 0.4-2.4 (1.8) |  | 0.9-2.8 (1.7) |  | 0.9-3 (1.7) |
|  |  |  |  |  |  |  |  |  |  |  |  |  |  |  |  |  |  |  |
